# A novel description of converting a gastric plication to sleeve gastrectomy for weight loss: A case report

**DOI:** 10.1016/j.ijscr.2021.105972

**Published:** 2021-05-12

**Authors:** Jonathan R. Chino, Garrett JonesDO, Amit B. Karmur, Robert Stowe, Barry Sanchez

**Affiliations:** a Community Memorial Health System, 147 N Brent. St, Ventura, CA 93003, United States of America; b Ventura County Medical Center, 300 Hillmont Ave, Ventura, CA 93003, United States of America

**Keywords:** Case report, Bariatric, Weight loss, Gastric plication, Sleeve gastrectomy

## Abstract

**Introduction:**

Bariatric surgery is a rapidly growing field with trends and standards of care changing more rapidly than most. Gastric plication was once an exciting novel procedure which showed promise, however it has fallen out of favor for other procedures such as the sleeve gastrectomy. Existing literature on the surgical conversion of a gastric plication to a sleeve gastrectomy does not provide specific details on the operative technique of this rarely encountered operation.

**Case report:**

In this case report we describe a morbidly obese female who presented weight gain after laparoscopic gastric plication and gastric banding. We provide the operative technique involved in conversion to a laparoscopic sleeve gastrectomy.

**Conclusion:**

This case provides specific operative technique with detailed media for the conversion of gastric plication to LSG.

## Highlights

• Patient returns to clinic after laparoscopic gastric plication and gastric band with failure to lose weight.
• Conversion of gastric plication to gastric sleeve is not a well-covered topic in literature.
• Laparoscopic gastric sleeve can be performed in the same operating of a gastric band removal and plication takedown.

## 1 Background

Bariatric surgery remains a pillar in the treatment of obesity. Restrictive procedures such as Laparoscopic Sleeve Gastrectomy (LSG) and gastric plication (GP) are two procedures that are commonly used initially in the surgical management of obesity. GP is a procedure that reduces the vertical dimensions of the stomach [1]. GP has shown to be as effective as other restrictive procedures in short-term weight reduction while being a less costly and less invasive option for early bariatric intervention for morbid obesity [2]. GP is more effective than lifestyle alone in weight loss management [3]. Other studies suggested that GP can be easily reversed and allows the option of performing future malabsorptive techniques in the situation of failure. This notion, however, has not been fully investigated.

While gastric plication has short-term benefits and is a low-cost procedure, it has several disadvantages. Its effectiveness in long-term weight-loss in comparison to LSG or other malabsorptive techniques is yet to be proven. Revision is often required within six years of plication [4]. The diameter of the stomach might return to pre-procedure levels within 12 months after gastric plication [5]. GP is inferior to other bariatric procedures in reducing comorbid conditions such as Diabetes Mellitus [6]. LSG is more effective than gastric plication in effective weight loss and decreasing postoperative length of stay; however, there is no difference in terms of comorbid condition reduction or adverse events [6].

The physiology, indications, and revision surgery techniques for failed plication have only been discussed in limited studies. To date, there has only been one case report that described a case of LSG revision after gastric plication. Coskun et al. discussed the indication for revision due to failure of the plication in terms of regaining weight and return of native size of the stomach, leading to LSG [7]. Goel et al. discuss two similar cases of reversal in the case a dilated stomach found distal to combined Laparoscopic Adjustable Gastric Banded Plication (LAGBP) [8]. We present a similar case, however, this case report will focus on describing the surgical technique performed during the revision as well as provide photos that can help guide surgeons that may not have much experience with reversal of this procedure that has not ever become widespread. Reoperations like this case can be quite complex. Case reports similar to this can serve as a foundation in creating a uniform surgical technique for future patients requiring conversion of gastric plication to LSG.

This case report has been reported in line with the SCARE Criteria [12].

## 2 Case presentation

The patient is a 38-year-old, morbidly obese female with hypertension, DMII, and a surgical history of Laparoscopic Gastric Plication (LGP) and Gastric Banding. At the time of evaluation, LGP history was unknown and not reported by the patient. She only recalled having had banding four years prior to presentation, and was unable to provide or obtain any documentation from the event. Her reported starting weight was 116 kg (BMI = 44 kg/m2), lowest post banding weight was 101 kg (BMI = 35 kg/m2), which was seen one year after the index procedure. Following her previous surgery, she did not visit a dietician, psychologist, or support group. The patient weighed 114 kg (BMI = 43 kg/m2) when she was evaluated for the first time in our clinic. She underwent the standard pre-operative evaluation required for gastric surgery at our center. This included a nutritional and diet evaluation, psychiatric evaluation, and analysis of family support for continued weight lost practice post procedure. Chest x-ray confirmed proper placement of the band. Conversion from Gastric Banding to a LSG or Roux-en-Y gastric bypass was recommended due to failure of prior bariatric surgical modalities, and the patient elected for LSG.

## 3 Operative description

**Fig. 1:**
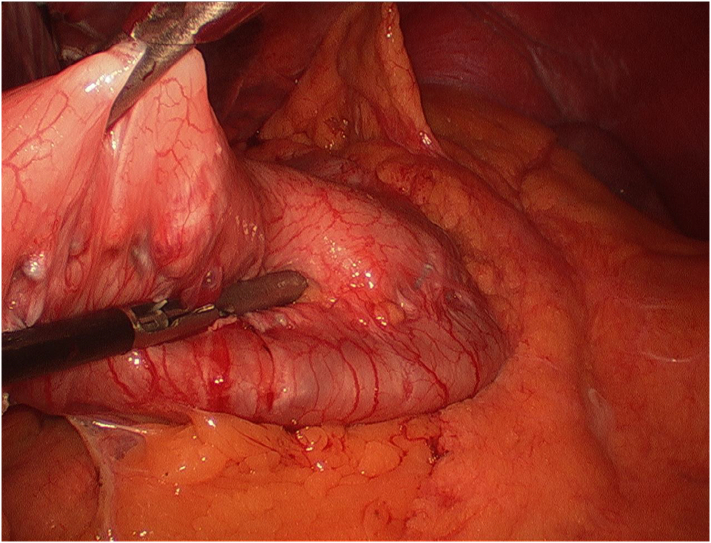
Gastric plication with adhesions noted upon inspection of greater curvature after removal of Gastric band.

**Fig. 2:**
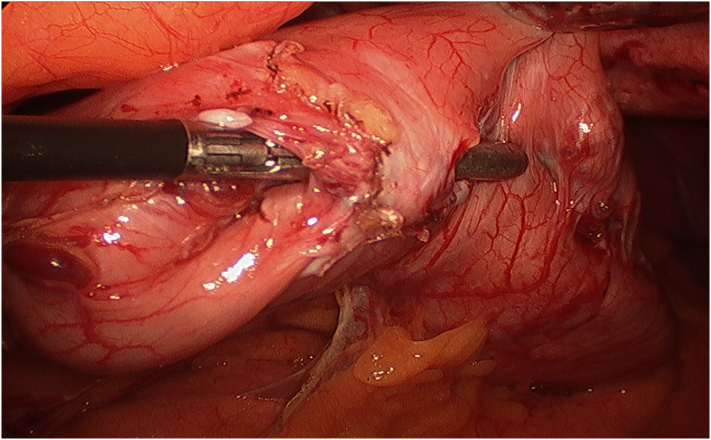
Release of plication from interrupted non-absorbable sutures with sharp dissection of gastric adhesions.

**Fig. 3:**
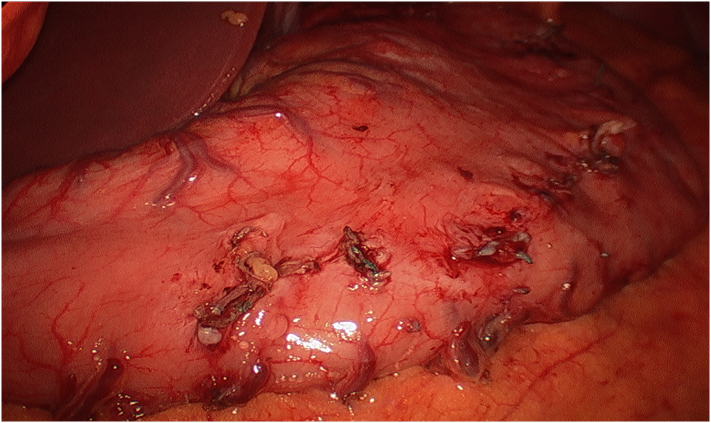
Completion of gastric plication release without any noted injury to the stomach. The green remnants of permanent suture can be seen.

**Fig. 4:**
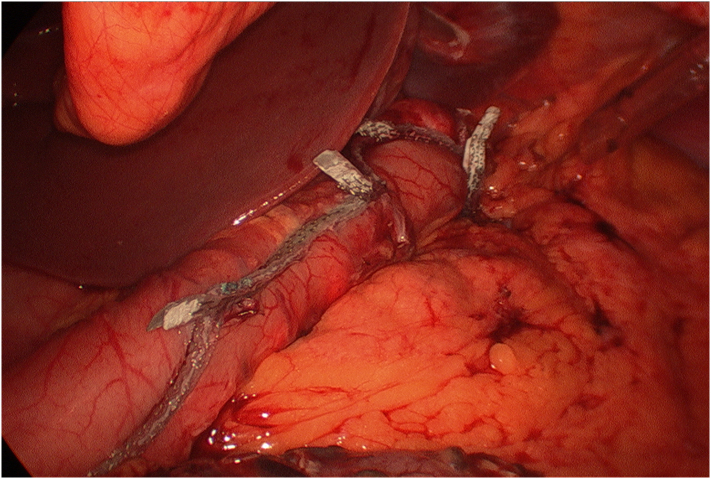
Successful completion of intended laparoscopic sleeve gastrectomy operation. Seamguard can be seen on the staple line.

After induction of anesthesia and surgical preparation, the operation was initiated similar to all of our LSG procedures. Veress needle technique was utilized to obtain pneumoperitoneum and entry to the peritoneal cavity via direct optical access. A 12-mm trocar was placed 15 cm inferior to the xiphoid process. Additional trocar placements included: a 15-mm trocar in the right upper quadrant; a 5-mm trocar in the left upper quadrant; a 5-mm trocar inferior to the left costal margin, midclavicular line; and a liver retractor probe was placed through a small incision in the subxiphoid area. Initial inspection demonstrated adhesions between the stomach and liver, which were divided. Gastric band tubing was noted to be intact, this was dissected out, and a capsulotomy was performed over the buckle of the tubing. The band was removed from around the stomach and the peritoneal cavity. While inspecting the greater curvature of the stomach in preparation for the sleeve gastrectomy, it was noted that a prior imbrication had been performed with what appeared to be non-absorbable, braided sutures. To allow the stomach to expand to its anatomical shape fully, the imbrication had to be released. This was completed with sharp dissection of the interrupted sutures and dissection of any adhesions that were present. When this was completed, with the stomach flat, a thorough evaluation of the stomach's anterior and posterior walls, including the lesser curvature, was done to identify any injuries to the stomach so that appropriate repairs might be made. There were no injuries noted. The sleeve gastrectomy portion of the case was completed in a standard fashion without complication. A 7-French closed suction drain was left in place along the staple line. She was discharged from the hospital on postoperative day one after negative fluoroscopic swallow evaluation and successful advancement of her diet, which is our standard of care for LSG. The drain was left in place and removed a few days later at clinic (Fig. 1, Fig. 2, Fig. 3, Fig. 4).

## 4 Discussion

During this patient's procedure, the discovery of gastric plication was unexpected due to the absent documentation of her prior bariatric surgical history. Without prior planning for take-down of the plication, the conversion of Gastric Band removal to sleeve gastrectomy was performed without perioperative or postoperative complications. Drain placement is not a standard step in our practice; however, it was left in this case given the extensive scar tissue that had to be removed to release the plication. We do not advocate for or against drain, placement and it should be decided on a case-by-case basis.

The issue of whether the index plication was performed without the patient knowledge or she simply did not recall correctly was discussed, however the procedure was performed in Mexico and we had no access to records. She was informed of the unexpected finding, and chose not to further pursue the matter. The patient has had moderate success in the primary outcome of weight reduction following her conversion. Follow-up visits at 1 and 6 months postoperatively showed the patient's reduced obesity class, from a pre-operative weight of 114 kg (BMI = 43 kg/m2) to a postoperative weight of 100 kg (BMI = 35 kg/m2), or a % extra weight loss (%EWL) of 25%. During her postoperative management, she was compliant with her dietary restrictions but did not continue to follow up with dietary support groups nor psychosocial interventions. The patient's 1-year follow-up evaluation is up coming, so the need for further bariatric intervention is yet to be seen. However, with the greater success levels of LSG compared to GP, there is hope that the patient will not require any more invasive procedures for failure or otherwise related to her obesity.

Goel, et al. as well Cornejo, et al. present similar procedures that first required reversal of GP. Cornejo, et al. in fact note that the extra mobility and visualization afforded by the Da Vinci robot system facility the fine technique required to release the suture and scar without damaging the stomach [9]. No drains were placed and all patients were released from the hospital soon after surgery without any complications. Zerrweck, et al. successfully converted thirty patients to either a LSG or Laparoscopic Gastric Bypass, providing additional support that the procedure can be performed safely [11]. We believe the media provided in this report will be helpful to any surgeons who are required to perform a similar reversal in the future, expected or not.

## 5 Conclusion

This case provides a foundation for future studies regarding the conversion of gastric plication to LSG. Gastric plication is a relatively new bariatric procedure. Therefore, more investigation must be done in regards to the successes and failures of techniques to convert to more invasive bariatric techniques. This case report is not intended to advocate for or against performing LGP, or its reversal as a standard of care. Based on a review of the literature, there is minimal description available of the technique used in the surgical conversion similar to what is present in this case report. This case is an exemplar that this procedure, even when encountered unexpectedly, can be performed safely and lead to successful results.

## Sources of funding

None.

## Ethical approval

Ventura County Medical Center (VCMC), the site from which all data was gathered, does not require IRB approval for sufficiently anonymous case reports.

## Consent

Informed consent was obtained from the patient for publication of this case report and accompanying images. A copy of the consent is available for review by the Editor-in-Chief of this journal on request.

## Registration of research studies

None.

## Guarantor

Jonathan R. Chino.

## CRediT authorship contribution statement

Jonathan R. Chino- Co-author

Garrett Jones- Co-author

Amit B. Karmur- Secondary surgeon (concept)

Robert Stowe-Co-author

Barry Sanchez- Primary attending surgeon (concept)

## Declaration of competing interest

None.
